# Are Youth Psychopathic Traits Related to Bullying? Meta-analyses on Callous-Unemotional Traits, Narcissism, and Impulsivity

**DOI:** 10.1007/s10578-016-0701-0

**Published:** 2016-12-10

**Authors:** Mitch van Geel, Fatih Toprak, Anouk Goemans, Wendy Zwaanswijk, Paul Vedder

**Affiliations:** Department of Child and Adolescent Studies, Leiden University, Wassenaarseweg, 2333 AK Leiden, The Netherlands

**Keywords:** Bullying, Psychopathy, Callous Unemotional, Narcissism, Impulsivity

## Abstract

In the current manuscript meta-analyses are performed to analyze the relations between three aspects of psychopathy in youth, Callous-Unemotional (CU) traits, Narcissism, and Impulsivity, and bullying behaviors. The databases PsycINFO, MEDLINE, ERIC, Web of Science and Proquest were searched for relevant articles on bullying and CU traits, Narcissism, or Impulsivity in youth under 20 years of age. Two authors each independently screened 842 studies that were found in the literature search. Two authors independently coded ten studies on bullying and CU (*N* = 4115) traits, six studies on bullying and Narcissism (*N* = 3376) and 14 studies on bullying and Impulsivity (*N* = 33,574) that met the inclusion criteria. Significant correlations were found between bullying and CU traits, Narcissism, and Impulsivity. These results were not affected by publication bias. Anti-bullying interventions could potentially benefit from including elements that have been found effective in the treatment of youth psychopathy.

## Introduction

Bullying is an intentional and repeated act of aggression against a relatively powerless victim [1]. Victimized youth have been found more likely to report psychosomatic complaints [2], non-suicidal self-injury [3], and even attempted suicide [4]. In a recent large scale study spanning 79 countries and more than 300,000 respondents, roughly 30% of adolescents reported being the victim of bullying [5]. Bullies may harass their victims because it helps them to achieve a position of dominance and popularity within the classroom [1, 6].

Youth scoring high on psychopathic traits may be especially prone to use bullying as an instrument to acquire dominance and popularity. Psychopathy is characterized by interpersonal, affective and behavioral dimensions [7], but a three dimensional approach wherein Callous-Unemotional (CU) traits, Narcissism, and Impulsivity are the core dimensions of psychopathy, has so far been the most influential in the study of youth and bullying [8–11]. CU traits refer to a lack of remorse and empathy, and a general uncaring attitude [12]. Narcissism concerns a sense of entitlement, the belief that one is more important than others, and a grandiose yet vulnerable self-image [13]. Impulsivity refers to a tendency to act on impulse and to not consider the long term consequences of actions [14]. Studies have repeatedly found that bullies are characterized by a lack of empathy towards their victims [15–17], a desire to look ‘cool’ and powerful [18], a need for dominating others [19–21], and dangerous and reckless behavior [22]. The constellation of these three traits is thought to define youth psychopathy [8–11], but each of these traits also has been suggested to be independently related to antisocial behaviors [11]. Studies indeed have found relations between bullying behaviors and CU traits [23, 24], Narcissism [11, 25] and Impulsivity [17, 26]. Increasingly, youth psychopathic traits are recognized as a risk marker for later conduct problems [27] and research on links between youth psychopathic traits and bullying is of particular importance because for youths scoring high on psychopathic traits etiological factors underlying their problem behaviors may differ from those typical of other youths who demonstrate problematic behaviors [28]. Furthermore, youth who score high on psychopathic traits are less likely to respond positively to typical interventions, and may be better helped by more individual and intensive approaches [28, 29]. As such, strong links between psychopathic traits and bullying may help to explain why so many typical anti-bullying interventions are ineffective [30, 31], and suggest whether bullies may benefit from interventions tailored to youth psychopathy.

Even though many studies have found links between psychopathic traits and bullying, not all studies have reported significant links [32, 33]. In the current study we conducted meta-analyses to statistically summarize the relations between bullying and CU traits, Narcissism and Impulsivity. A meta-analysis can be used to obtain a more robust effect size than individual studies [34]. Furthermore, by using a meta-analysis several statistics can be obtained to analyze to what extent publication bias has affected an overall effect size. Publication bias can emerge because journals may favor studies that report significant results. Studies that report non-significant results may not be published and end up in the ‘file drawers’ of researchers. If this consistently happens, a relation between two variables might be found significant because the existing null-findings have never been made available [34]. A final advantage of meta-analysis is that the sources of variance between studies can be statistically tested using moderator analyses. We choose to include mean age of the participants and the instruments used to measure bullying as moderators in the current meta-analyses. Based on the individual studies, we hypothesize significant relations between bullying and CU traits, Narcissism, and Impulsivity [10, 11, 17, 19, 23–27].

Psychopathy is thought to have a strong genetic component, and the extent to which genes affect behavior may differ for younger and older adolescents [35–39]. This clarifies why we study age as a moderator. However, a strong body of literature concerning the links between age, psychopathy and bullying is lacking; therefore we do not formulate a hypothesis for these analyses. With regard to instruments, we shall differentiate between self-reports and peer reports of bullying. Though many studies on bullying rely on self-reports, self-reports and peer reports of bullying tend to produce varying estimates of prevalence, and some even doubt the validity of self-reports on bullying [40]. Furthermore, because most personality questionnaires are self-reports, effect sizes in studies that use self-reports to measure bullying may be inflated due to shared method variance [41]. Due to shared method variance, we expect effect sizes between bullying and CU traits, Narcissism and Impulsivity to be higher in studies that used self-reports to measure bullying.

## Method

### Retrieval of Studies

**Fig. 1:**
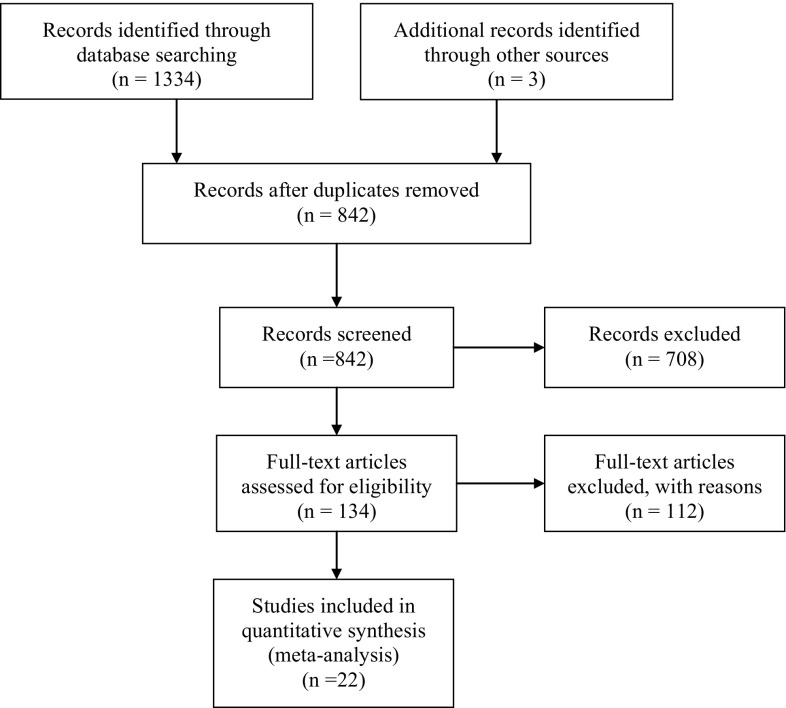
A flow diagram of the search results. *Adapted from* Moher et al. [68]

The databases PsycINFO, MEDLINE, ERIC, Web of Science and Proquest were searched using the key words “bully*”, “bullie*”, “peer victim*”, “peer harassment” or “school violence” and “dark triad”, “narcis*”, “mach*”, “callous”, “unemotional”, “callous-unemotional”, “psychopath”, “psychopathy”, “psychopathic”, “empath*”, “manipulation”, “grandiose*”, “impulsiv*”, “egocentricity”, “dirty dozen”, “dark tetrad”, “bistrategic controller”, “selfish*”, “remorseless*”, “defensive egotism”, “CU”, “APSD”, “ICU” or “antisocial process screening device” (May 7, 2015). No date limits were specified. The search terms “dark triad”, “dark tetrad”, and “dirty dozen” do not directly relate to psychopathic traits, but were added to avoid missing relevant sources with regard to Narcissism; however, these search terms did not provide articles that we otherwise would have missed. This search strategy yielded 1334 studies. After removing duplicates, 841 articles remained. A flow chart is included in Fig. 1. Two authors independently went through all the references to find studies for inclusion. Reference lists of retrieved studies were scanned for further articles. Studies were only included if they reported an effect size or provided enough information to compute an effect size. If there were several manuscripts that made use of the same dataset we only included one of these manuscripts to avoid having the same respondents in the meta-analysis multiple times. If manuscripts used the same data, those with more respondents were preferred over those with fewer respondents, more recent manuscripts were preferred over older manuscripts, and manuscripts that reported more useful effect sizes were preferred over those that included fewer. Several authors were mailed with a request to provide extra information if their articles did not report sufficient information for the computation of an effect size. This led to the inclusion of two articles [25, 42]. In the most common definition, bullying includes both repetition and a power imbalance [1], though some definitions only include repetition [43]. We included articles that focused on repetitive acts of violence against peers, though a power imbalance was not necessarily stated in the “Method” section of included articles; being able to repeatedly aggress against a peer already suggests a power imbalance. We only focused on Impulsivity, and not on self-control. Though self-control and impulsivity are strongly related concepts, self-control is a broader concept that encompasses more than impulsivity [44]. One article [44] used a self-control scale which included a subscale for impulsivity; from this article we coded the correlation between the impulsivity subscale with the bullying scale. Both community samples and clinical samples were included. Studies were only included if they compared a group of bullies to a group of non-involved children or adolescents, or if they provided a continuous measure (e.g., correlation) for the strength of concurrent associations between bullying and CU traits, Narcissism or Impulsivity. Studies that included participants aged 20 years or more were excluded. No lower age limit was used as exclusion criterion. Articles were excluded if they only provided data on prospective links between bullying and psychopathic traits. There were too few prospective studies to allow for a meaningful meta-analysis, and prospective links are often analyzed in a separate meta-analysis [2, 45]. From three longitudinal articles we decided to code the T1 links between bullying and psychopathic traits [10, 25, 46]; we chose to code the T1 data because this would be most comparable to the other included studies wherein respondents only reported on bullying once. The current meta-analysis focused on traditional bullying. We know of too few studies on cyberbullying and CU traits, Narcissism, and Impulsivity [47] to be meaningfully included in a meta-analysis. In total we found ten studies on bullying and CU traits [10, 11, 23, 24, 48–53] six studies on bullying and Narcissism [10, 11, 25, 33, 42, 52] and 14 studies on bullying and Impulsivity [10, 11, 17, 19, 26, 32, 33, 44, 46, 52, 54–57] that met our inclusion criteria. Most studies were written in English. One study was written in Spanish [32], one in Italian [33], and one study was written in Hungarian [50]. To minimize the potential impact of publication bias we chose to not only include peer reviewed articles, but also doctoral dissertations, book chapters, reports and research posters. Most included studies were published in peer reviewed journals (*K*_*studies*_ = 20, 90.9%), but we also included, two [26, 52] doctoral dissertations (*K*_*studies*_ = 2, 9.1%). The included studies were published between 1982 [19] and 2015 [51]—two studies appeared in print in 2016 [25, 48], but were already available online in 2015 and therefore included in the meta-analyses. An overview of all included studies is provided in Table 1.

**Table 1:** Included studies and their characteristics

Source	*N* (% female)	Age range	Context	Country	Bully measure	Personality scale	Included constructs
Ahmed and Braithwaite [54]	1401 (54.0)	Grades 4–7	Public and private schools	Australia	Single item (s)	Mix of junior impulsiveness scale and EASI-III temperament survey	IMP
Ando et al. [55]	2301 (49.8)	12–15 year	Junior high schools	Japan	11 items based on interviews and previous studies (s)	Weinberger adjustment inventory	IMP
Ang et al. [42]	809 (47.3)	9–16 year	Elementary and middle school	Singapore	7 items based on previous studies (s)	Narcissistic personality questionnaire for children-revised	NAR
Bjorkqvist et al. [19]	155 (43.9)	14–16 year	Comprehensive schools	Finland	Peer nominations (p)	Five point semantic differential	IMP
Bosworth et al. [57]	558 (54)	6th–8th grade	One middle school	USA	Five items from modified aggression scale	Four item scale developed for study	IMP
Chui and Chan [44]	365 (0.0)	10–17 year	Male only schools	Macua (China)	Illinois bullying scale (s)	Self-control scale	IMP
Ciucci et al. [23]	540 (52.6)	10–16 year	Middle school	Italy	Self-reports and peer nominations (s, p)^a^	Inventory of callous unemotional traits (ICU)	CU
Fanti et al. [24]	347 (49.3)	12–18 year	Middle and high schools	Cyprus	Student survey of bullying behavior revised (SSBB-R)	ICU	CU
Fanti and Kimonis [10]	1416 (50.1)	11–14 year	Middle schools	Cyprus	SSBB-R	ICU & antisocial processes screening device (APSD)	CU, NAR, IMP
Golmaryami et al. [48]	284 (54.2)	9–14 year	Public schools	United States	Modified participant role scale (p)	APSD	CU
Jolliffe and Farrington [17]	720 (47.8)	13–17 year	Comprehensive schools	United Kingdom	Several items based on previous literature (s)	Urgency component of UPPS	IMP
Low and Espelage [46]	1,232 (49.8)	10–15 year	Middle schools	United States	Illinois bullying scale (s)	Teen conflict survey	IMP
Martorell et al. [32]	108 (56.9)	9–15 year	Schools	Spain	BULL-S (s)	IVE-J Escala de impulsividad	IMP
King Meyer [26]	207 (56.5)	10–14 year	Middle schools	United States	Teen conflict survey (s)	Teen conflict survey	IMP
Muñoz et al. [49]	201 (50.2)	11–12 year	Secondary schools	England	Revised Olweus bully/victim questionnaire (RBVQ) (s)	ICU	CU
Nagy et al. [50]	117 (48.7)	12–14 year	Elementary schools	Hungary	Peer nomination (p)	ICU	CU
O’Brennan et al. [56]	24,345 (49.7)	Grade 4–12	Elementary, middle and high schools	United States	One item based on previous publications (s)	Four items developed for study	IMP
Panayiotou et al. [51]	91 (40.7)	5th–8th grade	Schools	Cyprus	RBVQ (s)	ICU	CU
Reijntjes et al. [25]	385 (51)	4th grade (mean age 10.4 year)	Primary schools	Netherlands	Bullying role nomination procedure (p)	Childhood narcissism scale	NAR
Sagone and Licata [33]	351 (50.4)	10–14 year	Public junior high schools	Italy	La mia vita a Scuola (s)	Il questionario di Adattamento interpersonale (QAI)	NAR, IMP
Sargeant [52]	315 (53.6)	11–14 year	Single secondary school	England	Olweus Bully-victim questionnaire (s)	Childhood narcissism scale, narcissistic personality inventory for children, UPPS, ICU	NAR, IMP, CU
Stellwagen and Kerig [11]	100 (38.0)	10–15 year	Inpatient psychiatric facility	United States	Participant roles in the bullying process (p)	APSD	CU, NAR, IMP
Viding et al. [53]	704 (47.0)	11–13 year	Secondary schools	England	Guess who measure of bullying (p)	ICU	CU

*s* self-report, *p* peer-report, *CU* Callous-Unemotional traits, *NAR* narcissism, *IMP* impulsivity

^a^This article included self and peer-reported measures of bullying. In the overall analysis self and peer-reported measures were averaged. In the moderator analysis this article was included as a peer-reported article after the self-reported correlation was removed

### Coding

If studies included multiple independent samples, these were entered in the analyses separately. If studies distinguished between different forms of bullying, for example physical or verbal bullying, the effect sizes of these studies were averaged prior to the analyses [17, 23, 26, 32, 33, 53, 55]. In one study that defined several aspects of Narcissism [52] and one study that defined several aspects of Impulsivity [56] we averaged these aspects prior to the analyses. Seventeen studies provided correlations as a measure of effect size. One study [19] provided a range of t-values referring to their analyses on impulsivity and bullying. In order not to overestimate the effects, we coded the lowest t-value provided into our meta-analysis, and transformed this t-value into a correlation. One study [26] provided standardized betas, which we transformed into correlations [58]. From two studies we coded means and standard deviations [17, 50], and from one study we used frequencies and incidence rates to derive odds ratios [56]. Effect sizes, context, sample size, gender distribution, the instruments used, the reporters, sampling method, participation rate, age ranges and mean age, and country where the study was performed were independently coded by two of the authors. Differences were resolved through discussion. Prior to discussion the rate of agreement was 87%.

### Analyses

All analyses were performed with Comprehensive Meta-Analysis 2.2 [59]. We analyzed the data using a random effects model, which is more appropriate for meta-analyses based on a literature search than a fixed effects model [60]. To address the problem of publication bias we used Orwin’s Fail-safe *N*, Kendall’s τ and the Duvall and the Tweedie Trim and Fill method. Orwin’s Fail-safe *N* estimates how many studies with non-significant results would be needed to reduce a meta-analytically obtained significant effect size to an effect size that has no practical significance. Using Kendall’s τ we calculated the association between variances and standardized effect sizes: a significant Kendall’s τ suggests that small studies with non-significant results tend not to be published, whereas a non-significant Kendall’s τ suggests the absence of such publication bias. The Duvall and Tweedie Trim and Fill method imputes effect sizes until the error distribution closely approximates normality, to provide a more unbiased estimate of the effect size than the observed estimate [34]. We used a moderator analysis to compare the effect sizes of studies that used peer reports and studies that used self-reports of bullying and we used meta-analytic regression to test the mean age of the participants in a sample as a moderator.

## Results

### Callous-Unemotional Traits and Bullying

**Fig. 2:**
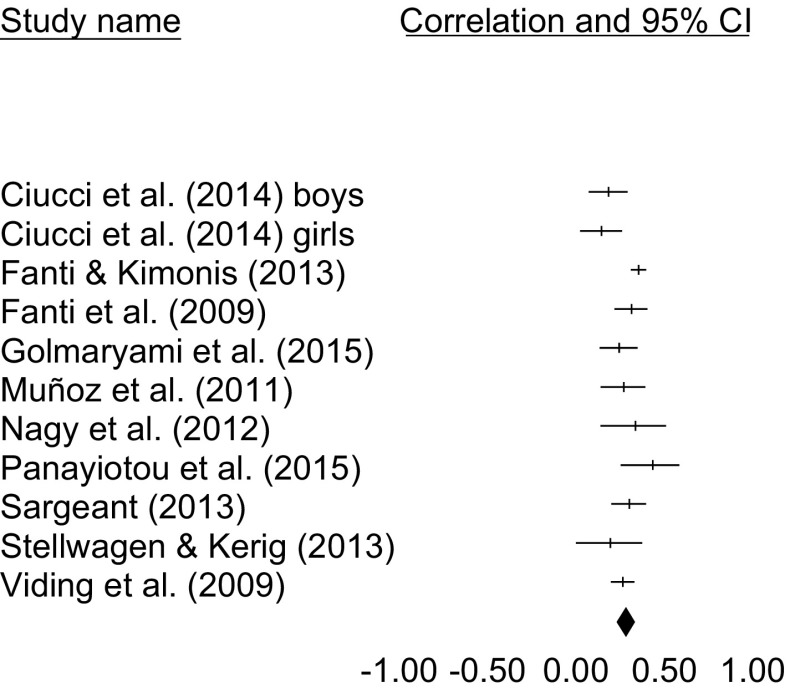
Forest plot for the studies included in the meta-analysis on CU traits and bullying

In a total of 11 samples (*N* = 4115), a significant relation was found between CU traits and bullying [*r* = .28, 95% CI 0.24–0.33], in a heterogeneous subset of studies [*Q*(10) = 22.003, *p* = .015, *I* ^2^ = 54.552]. A forest plot is included in Fig. 2. Removing one study at a time from the analyses provided estimates between *r* = .27 and *r* = .30, suggesting that the results are not strongly dependent on a single included study. Orwin’s Fail-safe *N* suggested that 57 studies with zero correlations would need to be found to reduce the obtained effect size to a trivial effect size of *r* = . 05. Kendall’s τ suggested that there were no significant relations between variances and effect sizes [τ = −0.072, *p* = .756]. Furthermore, the Duvall and Tweedie Trim and Fill method suggested that no effect sizes needed to be imputed to provide a more unbiased estimate. Taken together, these measures suggest that there was no strong influence of publication bias on the obtained effect size. One study [11] was based on an inpatient sample. We reran the meta-analysis with this study excluded; the results were similar to the originally obtained results [*r* = .29, 95% CI 0.24–0.34]. Using meta-analytic regression, age was not found to be a significant moderator in the relation between CU traits and bullying behaviors [*Q*(1) = 2.295, *p* = .130]. Articles that assessed bullying with peer nominations reported lower effect sizes [*k* = *5, r* = .23, 95% CI 0.17–0.28] than articles using self-reports to assess bullying [*k* = 6, *r* = .34, 95% CI 0.31–0.38], which was a significant interaction effect [*Q*(1) = 12.521, *p* < .001], though results were significant for both subsamples. The results are summarized in Table 2.

**Table 2:** Summary of the meta-analyses on bullying and CU traits, Narcissism, and Impulsivity

	*k*	*n*	*r*	*Q*	*I* ^2^	Orwin’s fail safe *N*
CU traits	11	4115	.28***	Q(10) = 22.003*	54.552	57
Narcissism	6	3376	.27*	Q(5) = 196.406***	97.454	32
Impulsivity	18	33,574	.25***	Q(17) = 152.681***	88.886	74

*p < .05. **p < .01. ***p < .001

### Narcissism and Bullying

**Fig. 3:**
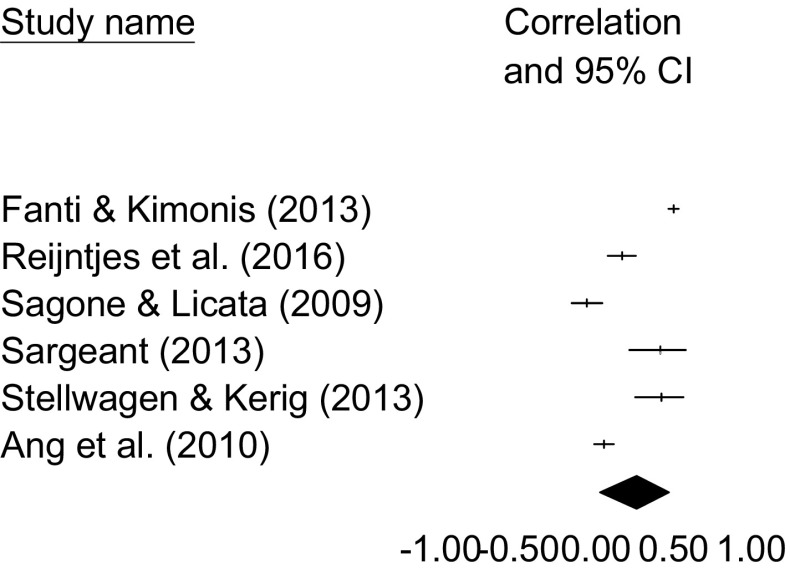
Forest plot for the studies included in the meta-analysis on Narcissism and bullying

In a total of six samples (*N* = 3376), a significant relation was found between Narcissism and bullying [*r* = .27, 95% CI 0.03–0.47], in a heterogeneous subset of studies [*Q*(5) = 196.406, *p* < .001, *I* ^2^ = 97.454]. A forest plot is included in Fig. 3. Removing one study at a time from the analyses provided estimates between *r* = .20 and *r* = .33. Orwin’s Fail-safe *N* suggested that 32 studies with zero correlations would need to be found to reduce the obtained effect size to a trivial effect size of *r* = .05. Kendall’s τ suggested that there were no significant relations between variances and effect sizes [τ = −0.00, *p* = 1.00]. Furthermore, the Duvall and Tweedie Trim and Fill method suggested that no effect sizes needed to be imputed to provide a more unbiased estimate, so that overall we conclude no strong influence of publication bias on the relation between Narcissism and bullying. One study [11] was based on an inpatient sample. When this study was removed, the correlation between Narcissism and bullying was no longer significant, though the effect size was similar to the one we obtained in the original results [*r* = .23, 95% CI −0.03 to 0.47]. There were only two studies on bullying and Narcissism that used peer-reports to study bullying [11, 25], and therefore we decided not to run moderator analyses to compare studies using peer reports or self-reports. Using meta-analytic regression, age was found to be a significant moderator in the relation between Narcissism and bullying [*Q*(1) = 72.985, *p* < .001], with the relation between Narcissism and bullying being stronger in studies wherein the mean age of the participants is higher. The results are summarized in Table 2.

### Impulsivity and Bullying

**Fig. 4:**
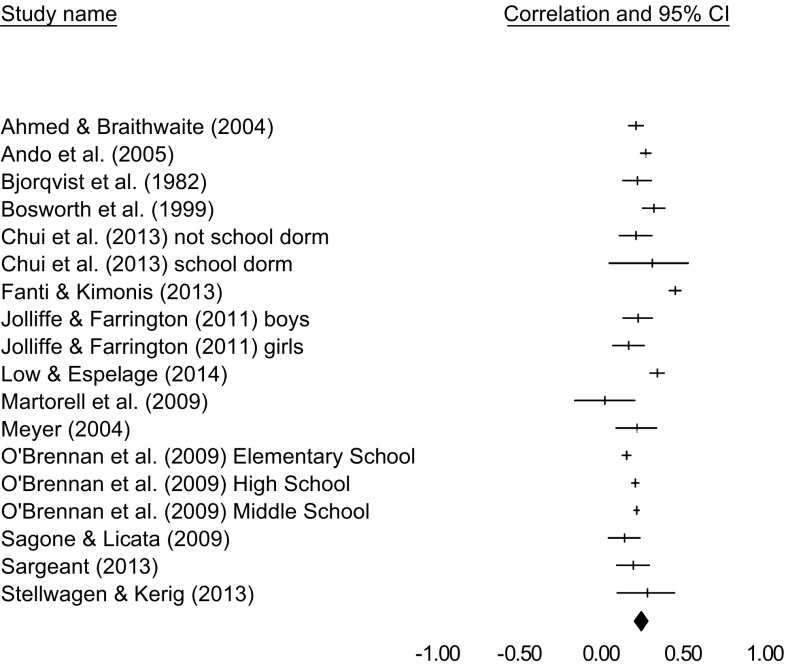
Forest plot for the studies included in the meta-analysis on Impulsivity and bullying

In a total of 18 samples (*N* = 33,574), a significant relation was found between Impulsivity and bullying [*r* = .25, 95% CI 0.21–0.29], in a heterogeneous subset of studies [*Q*(17) = 152.681, *p* < .001, *I* ^2^ = 88.886]. A forest plot is included in Fig. 4. Removing one study at a time from the analyses provided point estimates between *r* = .23 and *r* = .26, suggesting that the results are not strongly dependent on a single included study. Orwin’s Fail-safe *N* suggested that 74 studies with zero correlations would need to be found to reduce the obtained effect size to a trivial effect size of *r* = .05. Kendall’s τ suggested that there were no significant relations between variances and effect sizes [τ = 0.04, *p* = .82]. Furthermore, the Duvall and Tweedie Trim and Fill method suggested that no effect sizes needed to be imputed to provide a more unbiased estimate. Taken together, these measures suggest that there was no strong influence of publication bias on the obtained effect size. One study [11] was based on an inpatient sample. We reran the meta-analysis with this study excluded, the results were similar to the originally obtained results [*r* = .25, 95% CI 0.20–0.29]. Using meta-analytic regression, age was found to be a significant moderator in the relation between Impulsivity and bullying [*Q*(1) = 7.431, *p* = .006]. For older children relations between Impulsivity and bullying behaviors were stronger than for younger children. Because only two articles used peer nominations to analyze the relation between bullying and impulsivity [11, 19], we did not analyze differences between studies using peer reports and self-reports. The results are summarized in Table 2.

## Discussion

The current meta-analysis was meant to statistically summarize the relations between bullying and the three important characteristics of psychopathy in youth: CU traits, Narcissism, and Impulsivity. Significant and sizeable effect sizes were found between bullying and these three components of psychopathy, which support our hypotheses and confirm the results from most of the included individual studies; there are relations between psychopathic traits and bullying behaviors. We used several analyses to address potential publication bias, but we found no indication that publication bias had a strong effect on the obtained results. Though unpublished manuscripts with non-significant results might exist, it is unlikely that so many exist that they would nullify the significant associations reported.

In line with our hypothesis we found sizable and significant relations between CU traits, Narcissism, Impulsivity and bullying. The current meta-analysis is based on cross-sectional studies, and therefore we should be careful in terms of cause and effect reasoning, but some explanations for these results have been offered in the literature. Children and adolescents scoring high on CU traits are less sensitive to the fear and suffering of others, and are more likely to expect positive outcomes of aggression [61], which may explain a stronger inclination to bully others. Children and adolescents scoring high on Narcissism may bully others to maintain a sense of power and a grandiose self-image, or to gain entrance to a social group of antisocial yet popular peers [11]. Children who score high on Impulsivity may bully more because they tend to neglect long-term consequences [14] such as punishment by the teacher. They may bully because they feel that they have been provoked, or perhaps they ‘act without thinking’ when bullying others.

We found that the relations between bullying and Impulsivity and Narcissism were moderated by age, the relations being significantly stronger for older children. However, we found no age moderation for CU traits. CU traits are not necessarily stronger related to antisocial behavior than Narcissism or Impulsivity, but are a characteristic of children who show a relatively stable pattern of antisocial behavior in all stages of their development [12], which aligns well with the results of the current meta-analysis. Perhaps some degree of Narcissism and Impulsivity in adolescence is normative and not strongly related to bullying, whereas among older youth these traits may be more indicative of Psychopathy and therefore more strongly related to bullying.

Our hypothesis concerning self-reports and peer reports on bullying was confirmed, though we could only test this hypothesis for CU traits. We did not perform this moderator analysis for Impulsivity or Narcissism because there were only two studies on bullying and Impulsivity, and two studies on bullying and Narcissism that used peer reports to identify bullies. For the studies on CU traits and bullying we found that the effect sizes were higher for studies that used self-reports to measure bullying. We suspect that shared method variance [41] may be an explanation for this result. Even though the importance of peer reports have been argued [40], and the use of only self-reports may inflate effect sizes due to same method variance [41], many researchers still rely solely on self-reports to study bullying. The results of the current meta-analysis should not be taken to mean that self-reports are invalid; they may provide important information that peer reports do not [62]. However, the current meta-analysis suggests that peer reports are underused in the study of psychopathy and bullying in youth, and we agree that the best way to advance our knowledge of bullying is by considering self-reports and peer reports simultaneously [62].

In the current manuscript we ran meta-analyses on the independent correlations between CU traits, Narcissism, Impulsivity and bullying. However, psychopathy is commonly defined as a constellation of personality traits [7, 8], which goes beyond the independent correlations analyzed in the current manuscript. Following the approach presented in this paper, a youth would have to score high on all three traits to be a ‘psychopathic personality’. Scoring high on all three traits may relate to bullying in a different manner than scoring high on one trait only. It is possible that youth who are not ‘psychopathic personalities’ still score high on Narcissism or Impulsivity. We found too few studies to assess whether youth who score high on all traits differ in terms of bullying from youth who only score high on one trait. Nonetheless, in line with what has been suggested earlier [11] we found that CU traits, Narcissism, and Impulsivity were all correlated with bullying behaviors. This sheds new light on the personality traits that may underlie bullying, and stresses that in the study of youth psychopathy focusing on traits beyond CU traits may improve the prediction of antisocial behaviors [63].

The current meta-analysis is not without limitations. We could not differentiate between different forms of bullying, such as verbal or physical bullying. Furthermore, we did not include articles on cyberbullying in the current meta-analysis. Even though there may be links between psychopathic traits and cyberbullying [47], as of yet we found too few articles on cyberbullying and psychopathic traits to perform a meta-analysis. There were too few longitudinal articles to meaningfully include in a meta-analysis. Therefore we cannot conclude anything about the causality between bullying and psychopathic traits.

The current meta-analysis establishes links between psychopathic traits and bullying behaviors in youth. This may partly explain why two existing meta-analyses have found disappointing results about the effectivity of anti-bullying interventions [30, 31]; the violent behavior of youth scoring high on psychopathic traits is difficult to treat [64], and even though a meta-analysis suggests that successful treatment of psychopathy is possible, success is argued to be most likely at a high treatment intensity of four sessions a week for over a period of a year [29]; such high intensity treatment does not happen as a part of existing approaches to reduce bullying in the classroom. Even though generic approaches may not help, advances in knowledge about psychopathic traits [12] and insights gained from treating bullying as an evolutionary adaptation [65, 66] provide suggestions for useful practices to reduce bullying. Changing classroom norms to enable youth to acquire status through different means than aggressive displays [64] may be effective for those youth scoring high on Narcissism, preventive empathy training [12] may help reduce bullying among youth high on CU traits, and anger regulation [67] may reduce bullying among those youth scoring high on impulsivity.

## Summary

The current study used meta-analyses to analyze relations between youth psychopathic traits and bullying behaviors. Three aspects of youth psychopathy were included: CU traits, Narcissism, and Impulsivity. The main analyses revealed that youth scoring higher on CU traits, Narcissism, and Impulsivity also scored higher on bullying behaviors. Publication bias did not appear to have a strong influence on the obtained results. Moderator analyses suggested that the links between Narcissism or Impulsivity and bullying were stronger for older youth than for younger children. No such moderation was found for the link between CU traits and bullying. Though future research should establish why this is so, children scoring high on CU traits have been known to demonstrate a relatively stable pattern of antisocial behaviors. Though results were significant for both self-reports and peer reports, studies using peer reports suggested smaller effects sizes between CU traits and bullying than studies using self-reports. Same method variance could be an explanation for this result, and these results again underscore the necessity of using both peer and self-reports in the study of bullying. The current study further establishes psychopathic traits as a risk marker for youth behavior problems.

## References

[1] Salmivalli C (2010). Bullying and the peer group: a review. Aggres Violent Behav.

[2] Gini G, Pozzoli T (2013). Bullied children and psychosomatic problems: a meta-analysis. Pediatrics.

[3] Van Geel M, Goemans A, Vedder P (2015). A meta-analysis on the relation between peer victimization and adolescent non-suicidal self-injury. Psychiat Res.

[4] Van Geel M, Vedder P, Tanilon J (2014). Relationship between peer victimization, cyberbullying, and suicide in children and adolescents: a meta-analysis. JAMA Pediatr.

[5] Elgar FJ, McKinnon B, Walsh SD, Freeman J, Donnelly PD, de Matos MG (2015). Structural determinants of youth bullying and fighting in 79 countries. J Adolesc Health.

[6] Juvonen J, Graham S, Schuster B (2003). Bullying among young adolescents: the strong, weak, and troubled. Pediatrics.

[7] Hare RD (2003). Manual for the revised psychopathy checklist.

[8] Cooke DJ, Michie C (2001). Refining the construct of psychopathy: towards a hierarchical model. Psychol Assess.

[9] Fanti KA, Kimonis ER (2012). Bullying and victimization: the role of conduct problems and psychopathic traits. J Res Adolesc.

[10] Fanti KA, Kimonis ER (2013). Dimensions of juvenile psychopathy distinguish “bullies,” “bully-victims,” and “victims”. Psychol Violence.

[11] Stellwagen KK, Kerig PK (2013). Ringleader bullying: association with psychopathic narcissism and theory of mind among child psychiatric inpatients. Child Psychiat Hum Dev.

[12] Frick PJ, White SF (2008). Research review: the importance of callous-unemotional traits for developmental models of aggressive and antisocial behavior. J Child Psychol Psychiatry.

[13] Morf CC, Rhodewalt F (2001). Unraveling the paradoxes of narcissism: a dynamic self-regulatory processing model. Psychol Inq.

[14] Carver CS (2005). Impulse and constraint: perspectives from personality psychology, convergence with theory in other areas and potential for integration. Pers Soc Psychol Rev.

[15] Caravita S, Di Blasio P, Salmivalli C (2009). Unique and interactive effects of empathy and social status on involvement in bullying. Soc Dev.

[16] Gini G, Albiero P, Benelli B, Altoè G (2007). Does empathy predict adolescents’ bullying and defending behavior?. Aggress Behav.

[17] Jolliffe D, Farrington DP (2006). Examining the relationship between low empathy and bullying. Aggress Behav.

[18] Farrington DP (1993). Understanding and preventing bullying. J Crime Justice.

[19] Bjorqkvist KAJ, Ekman K, Lagerspetz K (1982). Bullies and victims: their ego picture, ideal ego picture and normative ego picture. Scand J Psychol.

[20] Pellegrini AD (2002). Bullying, victimization, and sexual harassment during the transition to middle school. Educ Psychol.

[21] Salmivalli C, Peets K, Rubin K, Bukowski W, Laursen B (2008). Bullies, victims, and bully-victim relationships. Handbook of peer interactions, relationships, and groups.

[22] Liang H, Flisher AJ, Lombard CJ (2007). Bullying, violence, and risk behavior in South African school students. Child Abuse Negl.

[23] Ciucci E, Baroncelli A, Franchi M, Golmaryami FN, Frick PJ (2014). The association between callous-unemotional traits and behavioral and academic adjustment in children: further validation of the Inventory of Callous-Unemotional Traits. J Psychopathol Behav.

[24] Fanti KA, Frick PJ, Georgiou S (2009). Linking callous-unemotional traits to instrumental and non-instrumental forms of aggression. J Psychopathol Behav.

[25] Reijntjes A, Vermande M, Thomaes S, Goossens F, Olthof T, Aleva L (2016). Narcissism, bullying, and social dominance in youth: a longitudinal analysis. J Abnorm Child Psych.

[26] *King Meyer S (2004) An examination of aggressive and prosocial behaviors among middle school students in a suburban school district. Dissertation, Wayne State University, Detroit

[27] Longman T, Hawes DJ, Kohlhoff J (2016). Callous–unemotional traits as markers for conduct problem severity in early childhood: a meta-analysis. Child Psychiat Hum Dev.

[28] Frick PJ, Ray JV, Thornton LC, Kahn RE (2014). Can callous-unemotional traits enhance the understanding, diagnosis, and treatment of serious conduct problems in children and adolescents? A comprehensive review. Psychol Bull.

[29] Salekin RT (2002). Psychopathy and therapeutic pessimism: clinical lore or clinical reality?. Clin Psychol Rev.

[30] Ferguson CJ, San Miguel C, Kilburn JC, Sanchez P (2007). The effectiveness of school-based anti-bullying programs. A meta-analytic review. Crim Justice Rev.

[31] Merrell KW, Gueldner BA, Ross SW, Isava DM (2008). How effective are school bullying intervention programs? A meta-analysis of intervention research. Sch Psychol Quart.

[32] Martorell C, González R, Rasal P, Estellés R (2009). Convivencia e inteligencia emocional en niños en edad escolar [Living together and emotional intelligence in school-age children]. Eur J Educ Psychol.

[33] Sagone E, Licata L (2009). Relazione tra adattamento interpersonale, disimpegno morale, bullismo e comportamento prosociale: una ricerca nella scuola media [The relationship among interpersonal adjustment, moral disengagement, bullying, and prosocial behavior: a study in junior high school]. G Psicol.

[34] Borenstein M, Hedges LV, Higgins JPT, Rothstein HR (2009). Introduction to meta-analysis.

[35] Larson H, Andershed H, Lichtenstein P (2006). A genetic factor explains most of the variation in the psychopathic personality. J Abnorm Child Psych.

[36] Taylor J, Loney BR, Bobadilla L, Iacono WG, McGue M (2003). Genetic and environmental influences on psychopathy trait dimensions in a community sample of male twins. J Abnorm Child Psych.

[37] Vernon PA, Villani VC, Vickers LC, Harris JA (2008). A behavioral genetic investigation of the Dark Triad and the Big 5. Pers Indiv Differ.

[38] Ferguson CJ (2010). Genetic contributions to antisocial personality and behavior: a meta-analytic review from an evolutionary perspective. J Soc Psychol.

[39] Bergen SE, Gardner CO, Kendler KS (2007). Age-related changes in heritability of behavioral phenotypes over adolescence and young adulthood: a meta-analysis. Twin Res Hum Genet.

[40] Cornell DG, Brockenbrough K (2004). Identification of bullies and victims: a comparison of methods. J Sch Violence.

[41] Hawker DS, Boulton MJ (2000). Twenty years’ research on peer victimization and psychosocial maladjustment: a meta-analytic review of cross-sectional studies. J Child Psychol Psychiatry.

[42] Ang RP, Ong EY, Lim JC, Lim EW (2010). From narcissistic exploitativeness to bullying behavior: the mediating role of approval-of-aggression beliefs. Soc Dev.

[43] Rose CA, Monda-Amaya LE, Espelage DL (2010). Bullying perpetration and victimization in special education: a review of the literature. Remedial Spec Educ.

[44] Chui WH, Chan HCO (2013). Association between self-control and school bullying behaviors among Macanese adolescents. Child Abuse Negl.

[45] Reijntjes A, Kamphuis JH, Prinzie P, Telch MJ (2010). Peer victimization and internalizing problems in children: a meta-analysis of longitudinal studies. Child Abuse Negl.

[46] Low S, Espelage D (2014). Conduits from community violence exposure to peer aggression and victimization: contributions of parental monitoring, impulsivity, and deviancy. J Couns Psychol.

[47] Eksi F (2012). Examination of narcissistic personality traits’ predicting level of internet addiction and cyber bullying through path analysis. Educ Sci Theory Pract.

[48] Golmaryami FN, Frick PJ, Hemphill SA, Kahn RE, Crapanzano AM (2016). The social, behavioral, and emotional correlates of bullying and victimization in a school-based sample. J Abnorm Child Psychol.

[49] Muñoz LC, Qualter P, Padgett G (2011). Empathy and bullying: exploring the influence of callous-unemotional traits. Child Psychiat Hum Dev.

[50] Nagy I, Pataky N, Szklenárik P, Körmendi A (2012). A rideg/erzeketlen vonasok jelenlete a zakatlas szerepkoreiben levo diakok kozott [The presence of callous/unemotional traits among students in different roles of bullying]. Psychiatr Hung.

[51] Panayiotou G, Fanti KA, Lazarou C (2015). Fearful victims and fearless bullies? Subjective reactions to emotional imagery scenes of children involved in school aggression. Personal Individ Differ.

[52] *Sargeant CC (2013) Examining the relationship between sources of self-concept and forms of aggression in adolescence. Thesis, University of Southampton

[53] Viding E, Simmonds E, Petrides KV, Frederickson N (2009). The contribution of callous-unemotional traits and conduct problems to bullying in early adolescence. J Child Psychol Psychiatry.

[54] Ahmed E, Braithwaite V (2004). Bullying and victimization: cause for concern for both families and schools. Soc Psychol Educ.

[55] Ando M, Asakura T, Simons-Morton B (2005). Psychosocial influences on physical, verbal, and indirect bullying among Japanese early adolescents. J Early Adolesc.

[56] O’Brennan LM, Bradshaw CP, Sawyer AL (2009). Examining developmental differences in the social-emotional problems among frequent bullies, victims, and bully/victims. Psychol Sch.

[57] Bosworth K, Espelage DL, Simon TR (1999). Factors associated with bullying behavior in middle school students. J Early Adolesc.

[58] Peterson RA, Brown SP (2005). On the use of beta coefficients in meta-analysis. J Appl Psychol.

[59] Borenstein M, Hedges LV, Higgins JPT (2005). Comprehensive Meta-analysis Version 2.2.

[60] Borenstein M, Hedges LV, Higgins J, Rothstein HR (2010). A basic introduction to fixed-effect and random-effects models for meta-analysis. Res Synth Methods.

[61] Pardini DA, Lochman JE, Frick PJ (2003). Callous/unemotional traits and social-cognitive processes in adjudicated youths. J Am Acad Child Adolesc Psychiatry.

[62] Gromann PM, Goossens FA, Olthof T, Pronk J, Krabbendam L (2013). Self-perception but not peer reputation of bullying victimization is associated with non-clinical psychotic experiences in adolescents. Psychol Med.

[63] Zwaanswijk W, Veen VC, Van Geel M, Andershed HA, Vedder P (in press) The relation between the bifactor model of the Youth Psychopathic traits Inventory and conduct problems in adolescence: variations across gender, ethnic background, and age. Psychol Assess 10.1037/pas0000407 27893226

[64] Harris GT, Rice ME, Patrick CJ (2006). Treatment of psychopathy: a review of empirical findings. Handbook of psychopathy.

[65] Ellis BJ, Del Giudice M, Dishion TJ, Figueredo AJ, Gray P, Griskevicius (2012). The evolutionary basis of risky adolescent behavior: implications for science, policy, and practice. Dev Psychol.

[66] Volk AA, Camilleri JA, Dane AV, Marini ZA (2012). Is adolescent bullying an evolutionary adaptation?. Aggress Behav.

[67] Larson J, Lochman JE (2003). Helping schoolchildren cope with anger.

[68] Moher D, Liberati A, Tetzlaff J, Altman DG, The PRISMA Group (2009). Preferred reporting items for systematic reviews and meta-analyses: the PRISMA statement. PLoS Med.

